# Comparison of the efficacy and safety of video-assisted thoracoscopic surgery with the open method for the treatment of primary pneumothorax in adults

**DOI:** 10.4103/1817-1737.37898

**Published:** 2008

**Authors:** Mohammad I Al-Tarshihi

**Affiliations:** *Division of Thoracic Surgery, King Hussein Medical Center, Royal Medical Services, Amman-Jordan*

**Keywords:** Pneumothorax, thoracotomy, video-assisted

## Abstract

**OBJECTIVE:**

To determine whether video-assisted thoracoscopic surgery is as effective as the traditional open method through axillary thoracotomy for the management of primary spontaneous pneumothorax in adults.

**MATERIALS AND METHODS:**

This retrospective study was conducted at King Hussein Medical Center in the period between March 2002 and March 2007. Eighty-two patients were included in this study. The patients were divided in two groups: group A, which included patients who underwent video-assisted thoracoscopic surgery; and group B, which included patients who underwent open technique through axillary thoracotomy. Efficiency of the procedure, operative time, postoperative complications, length of hospital stay, time to return to work and recurrence were compared between the two groups.

**RESULTS:**

There were 79 males (96.3%) and 3 females (3.7%) with a mean age of 23.7 ± 4.2 years for group A patients and 24.2 ± 4.6 years for group B patients (range 16–37 years). Forty-one patients (50%) underwent video-assisted thoracoscopic surgery (group A), and 41 patients (50%) underwent open surgical technique through axillary thoracotomy (group B). Postoperative complication occurred in 8 patients (19.3%) from among those who underwent open technique and 6 patients (14.6%) from among those who underwent thoracoscopic technique. There was no perioperative mortality in both groups. Postoperative pain, volume of blood loss, period of air leak and hospital stay were less in group A, although the operative time was less in group B.

**CONCLUSION:**

Video-assisted thoracoscopic surgery is an efficient and safe method for the treatment of patients with primary pneumothorax in the adults.

Pneumothorax, or air in the chest, is a phenomenon that has been recognized since ancient times. Hippocrates and Galen were aware of disease processes involving the pleural space.[1] Primary spontaneous pneumothorax is a relatively frequent condition. One of its most serious consequences is the high percentage of cases that require surgery (25-40%).[2] The thoracic surgeon is called to treat pneumothorax on regular basis. Controversy continues over the best method to treat pneumothorax. Video-assisted thoracic surgery (VATS) was introduced in 1990 and was quickly used as an approach strategy for many thoracic diseases, and this development has raised the controversy regarding the best treatment option for primary spontaneous pneumothorax.[1–3]

In this study, we compare the efficacy and safety of video-assisted thoracoscopic surgery with those of the open technique through axillary thoracotomy for the treatment of primary spontaneous pneumothorax in adults.

## Materials and Methods

This retrospective study was conducted at King Hussein Medical Center in the period between March 2002 and March 2007. Eighty-two patients were included in this study. Of these, forty-one patients underwent video-assisted thoracoscopic surgery (group A); 157 patients underwent open surgery through axillary thoracotomy, of which 41 patients (group B) were selected randomly using simple random sampling technique to ensure having two equal groups. There were clear indications to perform surgery for all the patients in both groups, and these included recurrent pneumothorax on the same side; bilateral pneumothorax; continuous air leak for more than 5 days; occupational indications, which included the pilots and people living in far areas. Chest X ray and chest CT scan were done for all patients prior to surgery to localize the presence and site of bullae. Single-lung ventilation was used. We performed lateral positioning of the patients. For the patients who underwent open technique through axillary thoracotomy, muscle-sparing thoracotomy was used; and the surgery consisted of extra-pleural approach - first, to separate the pleura from the chest wall; then, opening the pleura; identifying the bullae; doing bullectomy; and then apical pleurectomy, insertion of 28 French chest tube and closure. Regarding the VATS method, one optical and two working channels were used, doing bullectomy using a stapler; and apical pleurectomy using a subpleural needle (from DUPFNER Company / Germany); saline insufflation using an electrical pump, first to separate the pleura from the chest wall; and then for doing the pleurectomy, 28 French chest tube was inserted. All the patients were admitted to the thoracic surgery ward postoperatively, broad-spectrum prophylactic antibiotics and subcutaneous morphine were given to all patients for the first 24 h post-surgery and then parental non-steroidal anti-inflamatory were used later. Mankowski pain scale which ranged from zero to 10 was used to evaluate postoperative pain; the zero level indicates no pain, levels from 1 to 5 indicate mild pain and levels from 6 to 10 indicate moderate-to-severe pain, reaching the 10^th^ points indicating intolerable pain. All chest bottles were connected to negative suction of -20 K / pas postoperatively till the air leak stopped. The chest tubes were clamped for 24 h after being disconnected from the negative suction as an extra safe measure; hence, if any lung collapse in this period happened due to air leak, it could be simply reopened. The chest tube was removed if the lung was expanded fully as documented by the follow-up chest X ray. All the patients were discharged on the day of removal of chest tube. Follow-up in the clinic was done at 2 weeks, 1 month, 3 months and 6 months. Operative time, intraoperative blood loss, postoperative drainage of blood in the chest tubes, postoperative pain, postoperative complications, duration of air leak, length of hospital stay (pre- and postoperative), time to return to work and recurrence were compared between the two groups. Demographic, medical, intraoperative and postoperative data were collected for all patients. The student's t test was used for statistical study. Continuous variables were expressed as mean ± standard deviation, and categorical variables where expressed as percentages. The level of confidence was defined as a ‘*P*’ value of less than 0.05. StatsDirect statistical software version 2.6.5 was used to perform statistical analysis.

## Results

There were 79 males (96.3%) and 3 females (3.7%) with a mean age of 23.3 ± 4.2 years for group A patients and 24.2 ± 4.6 years for group B patients (range 16–37 years, *P* = 0.6202). Forty-one patients (50%) underwent video-assisted thoracoscopic surgery (group A), and 41 patients (50%) underwent open surgical technique (group B). The indications of surgery in group A were recurrent pneumothorax in 24 patients (58.5%), bilaterality in 6 patients (14.6%), persistent air leak for more than 5 days in 9 patients (22%) and occupational indication (pilots) in 2 patients (4.9%); while in group B, the indications for surgery were recurrent pneumothorax in 20 patients (48.8%), bilaterality in 5 patients (12.2%), persistent air leak for more than 5 days in 15 patients (36.6%) and 1 patient underwent surgery because he was living in far remote area (2.4%) (*P* = 1.000) [Table 1]. Regarding the operative time, it was 78.9 ± 6.2 min in group A (62–85 min), while in group B it was 28.8 ± 4.2 min (21–35 min) (*P* < 0.0001). Intraoperative and postoperative (as observed in the chest bottle) quantity of blood loss was 85.6 ± 26.2 ml (50-150 cc) in group A patients, while it recorded 243.9 ± 79.1 ml (100–500 cc) in group B (*P* < 0.0001). The postoperative pain measurements which were recorded by Mankowski pain scale showed that group A patients collected a score of 5.1 ± 0.8 (4–6 points), while in group B the measurements increased to 7.2 ± 1.1 (5-9 score)(*P* < 0.0001). Regarding the postoperative complications in group A, 5 patients (12.2%) suffered from intercostal neuralgia for around 3 months' period, and prolonged air leak postoperatively for 1 week occurred in 1 patient (2.4%); while in group B, 1 patient underwent reopening through posterolateral thoracotomy for bleeding during the immediate postoperative period, and the source was found to be left internal mammary artery iatrogenic injury, 2 patients (4.9%) had prolonged air leak for more than 5 days and 1 patient (2.4%) had recurrence of the pneumothorax during the first week postoperatively, and shoulder pain occurred in 4 patients (9.6%) [Table 2]. There was no perioperative mortality in both groups. Pre- and postoperative hospitalization days were 5.4 ± 0.7 days (4–7 days) in group A and 5.7 ± 0.6 days (5–7 days) in group B (*P* = 0.0167) [Table 3]. No recurrence was reported in both groups after the first week of surgery. All the patients were free of pain in both groups after 3 months of surgery, and 36 patients (87.8%) of group A were sent back to work after the first month follow-up; while in group B, only 21 patients (51.2%) returned to their work after the first month follow-up.

**Table 1:** Demographic data and indications of surgery in patients who underwent video-assisted thoracic surgery versus that of open bullectomy and apical pleurectomy

Characteristic	VAT (Group A)	Open (Group B)
Number	41	41
Age (Y)*	23.7 ± 4.2	24.2 ± 4.6
Sex (M/F)	39/2	40/1
Recurrence	24 (58.5%)	20 (48.8%)
Bilateralism	6 (14.6%)	5 (12.2%)
Persistent air leak	9 (22%)	15 (36.6%)
Occupational	2 (4.9%)	0 (0%)
Remote areas residence	0 (0%)	1 (2.4%)

VAT - Video-assisted thoracic

**Table 2:** Recorded postoperative complications

Complication	VAT (n = 41)		Open (n = 41)	
		
	Number	Percentage	Number	Percentage
Bleeding	0	0	1	2.4
Air leak	1	2.4	2	4.9
Early recurrence	0	0	1	2.4
Late recurrence	0	0.0	0	0.0
Intercostal neuralgia	5	12.2	0	0
Chronic shoulder pain	0	0.0	4	9.6
Mortality	0	0.0	0	0.0
Total	6	14.6	8	19.3

VAT - Video-assisted thoracic

**Table 3:** Comparison of the recorded data between VAT surgery and open surgery for the treatment of primary spontaneous pneumothorax

Recorded data	VAT	open	*P* value
Operative time	78.9 ± 6.2 min	28.8 ± 4.2 min	<0.0001
	(62-85) min	(21-35) min	
Blood loss	85.6 ± 26.2 cc	243.9 ± 79.1cc	<0.0001
	(50-150) cc	(100-500) cc	
Post operative pain	5.1 ± 0.8 score	7.2 ± 1.1 score	<0.0001
	(Mild to moderate)	(Moderate to severe)	
Hospitalization	5.4 + 0.7 days	5.7 + 0.6 days	0.0167
	(4 to 7) days	(5 to 7) days	

VAT - Video-assisted thoracic

## Discussion

Spontaneous pneumothorax is a disabling disorder that may present either in young and otherwise healthy patients (primary pneumothorax) or as a complication of an underlying lung disease (secondary pneumothorax). Since the 1960s clinical trials have led to opposing recommendations for management of spontaneous pneumothorax, an issue still under debate particularly for the management of the second episode.[4] Treatment options include tube thoracostomy with instillation of tetracycline, talc or other pleural irritants; mechanical pleural abrasions; total pleurodesis through thoracotomy; apical parietal pleurectomy through video-assisted thoracoscopic surgery (VATS); and apical lung wedge resection as an adjunct or solitary treatment.[5] We are using the protocol of doing bullectomy and apical pleurectomy in the young age groups rather than pleurodesis or mechanical abrasion, although it is time consuming, to give them the best chance of preventing recurrence, keeping the method of pleurodesis for the cases of secondary pneumothorax, as documented by many authors.[1] The most accepted surgical indications to treat pneumothorax either surgically using axillary thoracotomy or thoracoscopically are recurrence on the same side; pneumothorax on the contralateral side; persistent air leak for more than 5 days; special occupations, and these include the scuba divers and the airplane pilots and people living in remote areas far away from any medical institute;[1,2,4,5] although nowadays the period of air leak which is considered for surgery is more than 3 days, and tension pneumothorax is added as a new indication for surgery even if it was the first attack.[1] Axillary thoracotomy has been the classic surgical treatment of primary spontaneous pneumothorax, with many satisfactory results described in literature.[2] The introduction of VATS at the outset of the 1990s has made it the ideal treatment, reported to achieve good results right from its first published accounts. At present, it is the preferred approach on patients with primary spontaneous pneumothorax. Among its advantages are shorter stay in the hospital and less postoperative pain, factors which, however, have been compared correctly in very few studies.[3,4,6,7]

The results of our study showed a longer operative time in doing the VATS as compared to the open group, and this is due to the fact that we have only recently adopted application of thoracoscopic surgery at our institute in the last few years and due to the need of time to do pleural insufflation with saline using the subpleural needle; although the learning curve is becoming better with time. Freixinet *et al.*[2] reported a comparable operative time between the open and the thoracoscopic procedure, with a median operating time of 42 min for the VATS; and this can be explained by the longer experience this group had in doing thoracoscopic surgery.[2] Kim *et al.* and other authors[8] reported operative time comparable to that in our study in doing the VAT bullectomy and pleurectomy. Postoperative pain was less in group A as compared to group B, and this result is comparable with many other studies comparing the two groups,[5] although Freixinet *et al.*[2] reported the same level of postoperative pain in both groups. Intra- and postoperative blood loss either during surgery or after the operation was more in group B, and our explanation is that axillary thoracotomy is a limited incision and you can't view all the edges of the removed pleura well and this may cause continuous minimal bleeding postoperatively; while in Group A patients, we could view all the edges of pleura in a magnified view, controlling the bleeders in a very efficient way during surgery using the electrocautery or the argon beam. In addition, using the saline insufflation method to separate the pleura from the chest wall using the pleural needle will create a clear dissection plane and this will also decrease the blood loss, and this is comparable with some studies regarding this subject.[7] Freixinet *et al.*[2] reported almost no difference in the amount of blood loss between the two groups. We reported a case from our study from the group B who suffered active bleeding in the immediate postoperative period, with immediate re-thoracotomy through posterolateral incision; and the bleeding source was due to left internal mammary artery injury (LIMA) due to an iatrogenic injury during the stapler insertion. Group B patients suffered more postoperative pain and were demanding more analgesia postoperatively, and this is comparable with many other studies; although Kim *et al.*[8] reported minimal difference in analgesia requirements between the two groups, finding the axillary thoracotomy group requiring slightly more analgesics, Passlick *et al.*[9] reported a clear difference in analgesia requirements between the axillary thoracotomy and the VATS, documenting that axillary thoracotomy patients needed more analgesics. We reported five cases (12.2%) of intercostal neuralgia in patients who underwent VATS, while no cases were reported in patients who underwent axillary thoracotomy; and in all the cases, the neuralgia was around the camera port and this is attributed mainly to the use of 10 mm working channel for the camera and the 10 mm scope; so we recommend using the 5-mm scope for these patients. The complaints of these five patients resolved completely in 3 months.

Bertrand *et al.*[10] reported even a higher percentage of neuralgia at the operated site in people who underwent VATS, reaching 32% in his series; while Yim and Liu[3] reported a much lower percentage of intercostal neuralgia (0.37%). There was almost no difference in air leak complication postoperatively in both groups (group A = 2.4%; group B = 4.9%), and this is comparable with many other studies discussing this issue.[2–6,8,10,11] One patient (2.4%) from group B suffered from early recurrence after removal of the chest tube in the first week after surgery and was re-treated by insertion of the chest tube with tetracycline pleurodesis. No late recurrence in both groups was seen during the 6 months' follow-up period. Such a result with zero percent late recurrence rate is comparable with the result of Thomas *et al.*[11] for the open technique and superior to the results reported by Naunheim *et al.*, Yim and Ho, Inderbitzi *et al.* and many other authors,[11] and this may be attributed to our relative short follow-up period and relatively low number of patients involved in our study. Shoulder pain and limitation of its movement at site of surgery occurred only in patients who underwent axillary thoracotomy (9.6%), and this is attributed to the positioning of the patient during the procedure as a relatively hyper-abducted shoulder should be made in order to have access to the axillary thoracotomy; while in patients who underwent VATS, abduction of the shoulder was not needed and only some flexion of the shoulder over the anterior chest was sufficient to perform the procedure. Some other authors considered the shoulder pain as part of the complications related to surgery.[2,5,8,10–12] Many authors, including Massard *et al.*, Bertrand *et al.*, Dumont *et al.* and others,[11] reported less hospitalization days for the patients who underwent the VATS, although we reported a minimal difference of hospitalization days in favor of group A patients. The mortality rate was zero in both groups, which indicates the safety of these procedures, similar to many other studies that also reported a zero mortality rate.[2,4,5,8,12,13] There was a significant difference at the 1-month follow-up between the two groups, showing that most of the patients in group A returned to their work, while around only half of group B went back to their work at this time; and this is attributed to the incisional pain of the performed thoracotomy.

## Conclusion

Both VATS and axillary thoracotomy are equally efficient and safe methods for the treatment of spontaneous pneumothorax in the adult age groups. Although there were some differences in the postoperative complications, these complications were temporary and not life threatening. We prefer the VATS over the open method because the postoperative pain is less, hospitalization is less and returning back to normal activities is earlier.
